# Association of plasma tryptophan concentration with periaqueductal gray matter functional connectivity in migraine patients

**DOI:** 10.1038/s41598-021-04647-0

**Published:** 2022-01-14

**Authors:** Kinga Gecse, Dóra Dobos, Csaba Sándor Aranyi, Attila Galambos, Daniel Baksa, Natália Kocsel, Edina Szabó, Dorottya Pap, Dávid Virág, Krisztina Ludányi, Gyöngyi Kökönyei, Miklós Emri, Gyorgy Bagdy, Gabriella Juhasz

**Affiliations:** 1 Department of Pharmacodynamics, Faculty of Pharmacy, Semmelweis University, Nagyvarad ter 4, Budapest, 1089 Hungary; 2 SE-NAP2 Genetic Brain Imaging Migraine Research Group, Hungarian Brain Research Program, Semmelweis University, Budapest, Hungary; 3 Division of Nuclear Medicine and Translational Imaging, Department of Medical Imaging, Faculty of Medicine, University of Debrecen, Debrecen, Hungary; 4 Institute of Psychology, ELTE Eötvös Loránd University, Budapest, Hungary; 5 Department of Anesthesiology, Critical Care and Pain Medicine, Center for Pain and the Brain (PAIN Research Group), Boston Children’s Hospital, Harvard Medical School, Boston, MA USA; 6 Department of Pharmaceutics, Faculty of Pharmacy, Semmelweis University, Budapest, Hungary; 7 NAP-2-SE New Antidepressant Target Research Group, Hungarian Brain Research Program, Semmelweis University, Budapest, Hungary; 8 MTA-SE Neuropsychopharmacology and Neurochemistry Research Group, Hungarian Academy of Sciences, Semmelweis University, Budapest, Hungary

**Keywords:** Biomarkers, Neurological disorders, Neuroscience, Magnetic resonance imaging

## Abstract

Altered periaqueductal gray matter (PAG) functional connectivity contributes to brain hyperexcitability in migraine. Although tryptophan modulates neurotransmission in PAG projections through its metabolic pathways, the effect of plasma tryptophan on PAG functional connectivity (PAG-FC) in migraine has not been investigated yet. In this study, using a matched case-control design PAG-FC was measured during a resting-state functional magnetic resonance imaging session in migraine without aura patients (n = 27) and healthy controls (n = 27), and its relationship with plasma tryptophan concentration (TRP) was assessed. In addition, correlations of PAG-FC with age at migraine onset, migraine frequency, trait-anxiety and depressive symptoms were tested and the effect of TRP on these correlations was explored. Our results demonstrated that migraineurs had higher TRP compared to controls. In addition, altered PAG-FC in regions responsible for fear-cascade and pain modulation correlated with TRP only in migraineurs. There was no significant correlation in controls. It suggests increased sensitivity to TRP in migraine patients compared to controls. Trait-anxiety and depressive symptoms correlated with PAG-FC in migraine patients, and these correlations were modulated by TRP in regions responsible for emotional aspects of pain processing, but TRP did not interfere with processes that contribute to migraine attack generation or attack frequency.

## Introduction

Migraine attack, which is characterised by moderate or severe throbbing headache accompanied by nausea, vomiting, sensitivity to light and sound, and worsening during physical activity, is considered a brain state with altered excitability^1^ with a high economic burden^2^. However, the “migraine brain” seems to be hypersensitive to sensory stimuli not just during attacks, but also between them^3,4^. For example, in headache-free periods migraineurs showed decreased habituation to auditory, visual and pain related stimuli during repetition^5^ and lower sensory threshold for several sensory modalities^1^. Augmented pain-induced activation in cognitive pain processing regions^6,7^ and increased cortical excitability in the visual network^8^ were also demonstrated. In addition, enhanced emotional information processing can be detected interictally in migraineurs signalling an increased sensitivity to psychosocial stressors^9,10^. Although, the exact mechanism of the hypersensitive reaction of the migraine brain is not fully understood, one potential brain area that might contribute to this phenomenon is the periaqueductal gray matter (PAG)^11^.

The PAG is situated in the midbrain with rich bidirectional connections to the ascending sensory pathways and the descending cortical and limbic modulatory pathways. Its main task is to integrate the ascending and descending information, and to set the optimal salience level of pain, anxiety and autonomic functions for promoting survival^12^. The PAG, as a key region of pain modulating pathways, is suggested to play a crucial role in migraine pathogenesis^13,14^. Several studies demonstrated PAG activity during spontaneous and induced migraine attacks that persisted even after successful treatment^11,15,16^. In addition, PAG has increased resting-state and pain induced functional connectivity with regions of nociceptive and somatosensory processing pathways in migraine patients and these connections showed association with headache frequency^17,18^. PAG functional connectivity with top-down pain modulating prefrontal cortical and limbic areas were decreased compared to non-migraineurs^19^ in these studies emphasising its potential role in sensory hypersensitivity in migraine. Recent studies also demonstrated that effective treatment of migraine^19^ and other chronic pain conditions^20,21^ could significantly modulate the functional connectivity of PAG. Beside pain modulation, PAG orchestrates passive and active defensive behaviour, “flight or fight” responses which at pathologic expression could lead to anxiety and depression^22,23^. Indeed, migraine is highly comorbid with anxiety and depression^24,25^ and they have shared biological processes^26^ including the tryptophan metabolic pathway and its effect on PAG.

L-tryptophan is an essential amino acid and thus its plasma concentration and tissue uptake are dependent on the quantity and quality of dietary intake. Transportation of tryptophan from the plasma to the brain is dependent on the large neutral amino acid transporter where plasma tryptophan competes with other large neutral amino acids (LNAAs) for uptake^27^. Therefore, depletion of brain tryptophan can be elicited by drinking tryptophan free, LNAA containing drinks which can induce intense headache, nausea and photophobia in migraine patients^28^, reduced pain threshold and heat tolerance in healthy people^29^, acute exacerbation of depressive symptoms in previously depressed patients^30^, and exacerbation of anxiety and panic symptoms in panic patients^31^. Furthermore, the daily dietary intake of tryptophan influences migraine attack frequency: susceptible people who consume relatively less tryptophan per day have an increased risk for developing migraine^32^. In addition, higher dietary tryptophan resulted in less depression, irritability and decreased anxiety in healthy adults^33^.

Although, the exact mode of action of low tryptophan is not clearly understood, it is well known that brain serotonin synthesis depends on plasma tryptophan availability and many of the acute tryptophan depletion (ATD) effects were associated with low serotonin neurotransmission^34^. However, only a fraction of tryptophan is converted to serotonin and the majority is metabolised through the “kynurenine shunt” that produces several other neuroactive metabolites^35^. Both the serotonin and kynurenine tryptophan metabolic pathways are involved in migraine pathophysiology through complex neuronal networks^36–38^. Furthermore, it has been demonstrated that neuronal transmission of PAG and its effect on pain and emotion processing could be modulated by tryptophan metabolic pathways. For example, different serotonergic receptor activation or blockade in PAG modulated pain perception and defensive behaviour^39–41^, while kynurenine injection into PAG elicited anxiolytic effect and inhibited periaqueductal gray cell activity by glutamatergic blockade^42,43^. Thus, based on these observations it can be hypothesised that PAG functional connectivity alterations in migraine might be related to tryptophan plasma concentration.

Although, some studies have investigated blood tryptophan concentration in migraine, the results are controversial so far reporting both higher and lower tryptophan concentrations in migraineurs compared to controls^36,44^. Furthermore, to the best of our knowledge, the effect of plasma tryptophan concentration on PAG functional connectivity in migraine has not been investigated yet. Therefore, our aim in this study was to investigate plasma tryptophan concentration and its effect on PAG intrinsic functional connectivity during resting-state fMRI in migraine without aura patients and healthy controls. In addition, we assumed that plasma tryptophan concentration also alters the relationship between PAG connectivity and age at migraine onset, migraine frequency, trait-anxiety and depressive symptoms.

## Results

### Descriptives and self-report data

Participants characteristics are shown in Supplementary Table S1. There was no difference between migraine and healthy group in age. In the study, a sex matched case control design was used 27–27 migraineurs and controls with 21 female and 6 male participants in each groups. 77.8% of the participants was female, which ratio is in accordance with the migraine prevalence sex distribution^45^. There was no difference between migraine and control group regarding trait-anxiety and depressive symptoms scores.

### Plasma tryptophan levels

Total tryptophan concentration in plasma was significantly higher in migraine group compared to healthy controls in two separate blood samples (BS) (1. BS mean [µg/ml] ± SD migraine: 9.32 ± 2.88; control: 7.12 ± 3.52, U = 136, p = 0.002; 2. BS mean [µg/ml] ± SD migraine: 9.07 ± 2.66, control: 7.52 ± 2.27, t(2.21) = 4.78, p = 0.032). There were no significant differences in tryptophan concentration regarding sex (1. BS mean [µg/ml] ± SD males: 8.12 ± 3.47; females: 8.19 ± 3.40, U = 188, p = 0.704; 2. BS mean [µg/ml] ± SD males: 7.88 ± 2.48, females: 8.41 ± 2.61, t(48) = 0.604, p = 0.549). In the first blood sample there is a significant correlation between tryptophan concentration and age (rho = 0.368, p = 0.010), but there is no significant correlation in the second blood sample (rho = 0.173, p = 0.228).

The mean tryptophan level was significantly higher (*F*(1,63) = 6.82, p = 0.012) in migraine patients (mean[µg/ml] ± SD: 9.16 ± 2.35) compared to healthy controls (mean [µg/ml] ± SD: 7.46 ± 2.53) after correction for age, sex and plasma LNAA level. Plasma tryptophan concentration positively correlated with trait anxiety (r = 0.410, p = 0.034) and depressive symptoms (r = 0.392, p = 0.043) in the migraine group, but they did not show correlation in healthy controls (trait anxiety: r = −0.078, p = 0.698; depressive symptoms: r = −0.056, p = 0.781). Migraine attack frequency (rho = −0.059, p = 0.770) and the age at migraine onset (r = 0.127, p = 0.528) did not correlate with plasma tryptophan concentration.

### Resting-state fMRI

#### Intrinsic functional connectivity of PAG

Results of intrinsic functional connectivity of left and right side of PAG in the total study population containing 54 subjects are shown in Supplementary Table S2. Both seed regions demonstrated significant positive functional connectivity with nearby brainstem structures and thalamus, right PAG showed additional positive functional connectivity with cerebellum. No significant negative correlations were detected in our sample. The results of the extended sample (64 subjects) are also shown in Supplementary Table S6.

#### Intrinsic functional connectivity of PAG comparing migraine patients with healthy controls

In migraine and control group comparison, significantly increased functional resting-state connectivity (Peak-T value = 4.09 at cluster-level p_FWE_ < 0.05, cluster size: 285 voxels) was revealed between left PAG and left postcentral gyrus in migraine patients compared to controls. The region of the postcentral gyrus that showed increased connectivity with the left PAG corresponds to the head and neck area of sensory homunculus. Right PAG showed increased functional connectivity with left orbital part of superior frontal gyrus (Peak-T value = 6.65 at cluster-level p_FWE_ < 0.05, cluster size: 137 voxels) in migraine patients compared to controls. There was no significantly increased functional resting-state PAG connectivity in controls compared to migraineurs.

#### Tryptophan effect on intrinsic functional connectivity of PAG: effect of diagnosis

Tryptophan did not show main effect on PAG intrinsic functional connectivity in the whole study population (n = 54). Meanwhile, tryptophan showed diagnosis dependent association with PAG intrinsic functional connectivity. Namely, when migraine group was compared to the control group there was a significant difference between the plasma tryptophan concentration effect on left PAG intrinsic functional connectivity with middle and occipital gyri and fusiform gyrus and left cerebellum (Table 1). Tryptophan concentration also showed significantly different effect on right PAG intrinsic functional connectivity with left fusiform gyrus, left superior and middle occipital gyrus when migraine patients were compared to controls (Table 1).

**Table 1:** Significantly different association between PAG intrinsic functional resting-state connectivity and plasma tryptophan concentration in migraine patients compared to controls.

Region	Cluster size (voxel)	Peak F-value	MNI coordinates (x y z)		
**Left PAG**					
R superior occipital gyrus	1763	26.26	26	−94	20
R middle occipital gyrus		20.75	32	−80	6
L superior occipital gyrus	1267	23.17	−14	−96	24
L middle occipital gyrus		22.19	−26	−86	16
L fusiform gyrus	1244	25.18	−44	−70	−18
L cerebellum IV–V		22.40	−6	−64	−6
R fusiform gyrus	176	19.69	30	−46	−16
**Right PAG**					
L fusiform gyrus	698	19.77	−36	−80	−14
L superior occipital gyrus	287	25.10	−20	−96	18
L middle occipital gyrus		18.22	−26	−88	16

Significance threshold was cluster-level p_FWE_ < 0.05 including at least 20 contiguous voxels. Results are corrected for age, sex and LNAA concentration.

*R* right, *L* left, *MNI* Montreal Neurological Institute.

### Correlation of plasma tryptophan concentration with intrinsic functional connectivity of PAG in migraine patients

**Figure 1:**
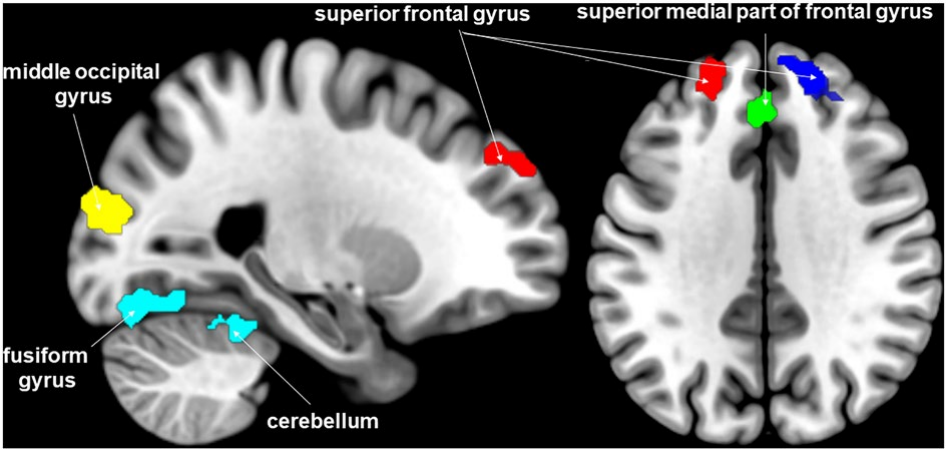
Brain clusters where intrinsic functional connectivity with PAG were significantly associated with plasma tryptophan concentration in migraine patients. Secondary cluster-level threshold p_FWE_ < 0.05. For visualization, the image file of significant clusters was downloaded from SPM12 and added as overlay on the MNI 152 template brain in MRIcroGL program (http://www.mccauslandcenter.sc.edu/mricrogl/).

Separately analysing the migraine group (Table 2 and Fig. 1), plasma tryptophan concentration positively correlated with the strength of intrinsic functional connectivity between both side of PAG and the superior and superior medial part of frontal gyrus. Significant negative correlations were found between plasma tryptophan concentration and intrinsic functional connectivity of left PAG with left and right fusiform gyrus, right cerebellum, both side of middle part of occipital gyrus and left superior occipital gyrus. Furthermore, significant negative correlation was found in the case of right PAG with left fusiform gyrus.

**Table 2:** Significant association between PAG intrinsic functional resting-state connectivity and plasma tryptophan concentration in migraine patients.

Region	Cluster size (voxel)	Peak T-value	MNI coordinates (x y z)		
**Left PAG**					
*Negative correlation*					
L fusiform gyrus	917	−8.36	−32	−48	−18
R fusiform gyrus	292	−5.99	28	−44	−18
R cerebellum VI		−5.00	34	−44	−26
L middle occipital gyrus	236	−4.87	−24	−84	14
L superior occipital gyrus		−4.11	−20	−96	18
R middle occipital gyrus	184	−4.61	42	−84	20
*Positive correlation*					
L superior frontal gyrus	162	6.04	−18	48	36
R superior frontal gyrus	162	5.58	20	44	32
R superior medial part of frontal gyrus		4.70	10	54	36
L superior medial part of frontal gyrus	148	4.91	−2	−36	32
**Right PAG**					
*Negative correlation*					
L fusiform gyrus	322	−7.25	−36	−36	–24
*Positive correlation*					
R superior frontal gyrus	297	7.11	20	44	32
R superior medial part of frontal gyrus		4.74	8	56	38
L superior frontal gyrus	170	5.98	−20	50	36
L superior medial part of frontal gyrus		5.00	−2	34	34

Significance threshold was cluster-level p_FWE_ < 0.05 including at least 20 contiguous voxels. Results are corrected for age, sex and LNAA concentration.

*R* right, *L* left, *MNI* Montreal Neurological Institute.

Separately analysing the control group, there were no significant associations between the plasma tryptophan concentration and functional intrinsic connectivity of PAG.

### Correlations of PAG intrinsic functional connectivity with migraine indicators, trait anxiety and depressive symptoms

The frequency of migraine attacks and the age of migraine onset significantly correlated with connectivity of PAG, but tryptophan concentration did not affect these associations (Supplementary Table S3).

Trait–anxiety level showed significant positive correlation with connectivity of left PAG and left middle frontal gyrus (Peak-T value = 5.33 at cluster-level p_FWE_ < 0.05). In case of right PAG, trait-anxiety also positively correlated with connectivity of right PAG and left middle (Peak-T value = 5.73 at cluster-level p_FWE_ < 0.05) and superior medial frontal gyrus (Peak-T value = 5.09 at cluster-level p_FWE_ < 0.05). After correction for plasma tryptophan concentration (also corrected for LNAA), trait-anxiety level did not show any significant correlation with PAG connectivity (Supplementary Table S3).

Depressive symptoms significantly positively correlated with connectivity of PAG and middle frontal gyrus (Peak-T value = 6.65 at cluster-level p_FWE_ < 0.05). In addition, a negative correlation with connectivity of right PAG and right fusiform gyrus (Peak-T value = −4.89 at cluster-level p_FWE_ < 0.05) and parahippocampal gyrus (Peak-T value = −4.14 at cluster-level p_FWE_ < 0.05) was found. After correction for plasma tryptophan concentration (also corrected for LNAA) there was no significant correlation between depressive symptoms and connectivity of PAG with middle frontal gyrus, fusiform gyrus or parahippocampal gyrus. However, depressive symptoms negatively correlated with left PAG and left precuneus (Peak-T value = −4.44 at cluster-level p_FWE_ < 0.05), right middle cingulum (Peak-T value = −4.08 at cluster-level p_FWE_ < 0.05) connectivity after correction for plasma tryptophan concentration (Supplementary Table S3).

To test the potential confounding effect of correlation between plasma tryptophan concentration and trait anxiety/depressive symptoms in migraineurs we re-tested the plasma tryptophan concentration correlation with PAG connectivity after correction for trait-anxiety level and depressive symptoms, see Supplementary Table S4.

In healthy controls, trait-anxiety level and depressive symptoms showed no correlation with PAG-FC.

## Discussion

Our study demonstrated an elevated plasma tryptophan concentration in episodic migraine patients without aura that modulated PAG intrinsic functional connectivity only in migraineurs but not in healthy controls. In addition, plasma tryptophan concentration also modified the relationship between PAG intrinsic functional connectivity and trait-anxiety and depressive symptoms in migraine patients.

### Increased plasma tryptophan concentration in migraine

In this study, we found elevated plasma tryptophan concentration during two independent blood sampling in interictal episodic migraine patients without aura compared to healthy controls. This finding is in concordance with a previous study of Alam et al.^46^, who demonstrated increased tryptophan concentration in episodic migraine, only in patients without aura. Another study showed trend level increase in plasma tryptophan concentration in migraine with or without aura patients^47^. Elevated serum tryptophan concentration and kynurenine pathway metabolites’ abnormalities are also present in chronic migraine^36^. Nonetheless, opposite results, namely lower serum tryptophan concentration and the role of serotonin metabolism, have also been reported in migraine without aura^44^.

Accumulating data suggest that the migraine attack itself can contribute to the elevated plasma tryptophan concentration in several ways^48,49^. The stress effect of the approaching or repeated attacks can increase cortisol secretion which will in turn increase proteolysis and plasma tryptophan concentration^35,49^. In general, decreased protein and increased carbohydrate intake, or fasting are able to increase the free plasma tryptophan concentration and promote the tryptophan uptake to the brain^35,48^. These processes can enhance coping with stress by increasing serotonin synthesis. However, prolonged stress eventually will shift the tryptophan metabolism towards the “kynurenine shunt” that might contribute to brain hypersensitivity and maladaptive stress response in migraine patients^35,38,50^.

In our study, a significant positive correlation was found between plasma tryptophan concentration and trait-anxiety and depressive symptoms, but only in the migraine group. Thus, specifically migraineurs showed increased sensitivity to tryptophan plasma concentrations, as can be seen in patients with depression^51^. In line with the acute tryptophan depletion literature^34,51^, in the control subjects we have not found any correlation between plasma tryptophan concentration and trait-anxiety and depressive symptoms. Regarding the positive direction of association between plasma tryptophan concentration and trait-anxiety and depressive symptoms in migraineurs, our finding may seem contradictory to the widely accepted theory, that acute tryptophan depletion induced decrease in serotonin concentration contributes to low mood and probably to anxiety^34^. However, our participants’ plasma tryptophan concentration was in the physiological range, during the experiment they followed their regular diet without any manipulations (depletion or supplementation). In addition, our migraine patients had no psychiatric disorders, and showed similar trait-anxiety and depressive symptom scores as the control participants. Thus, in migraineurs we could not exclude that plasma tryptophan elevation might be a part of a self-remediation process, elicited either by stress or by changes in diet, as discussed above.

Interestingly, we have not found significant correlation between plasma tryptophan concentration and age at migraine onset or migraine frequency despite that migraine patients thought to be sensitive to tryptophan intake^32^ and depletion as well^28^. However, tryptophan supplementation as a promising migraine treatment failed in previous studies^52,53^ which may support our observation that tryptophan concentration is associated with the emotional symptoms of migraine patients but does not interfere with processes that contribute to attack generation or attack frequency. Indeed, L-tryptophan administration alone cannot exert analgesic property^54^.

### Tryptophan modulates PAG intrinsic connectivity in migraine

Our study was the first to investigate tryptophan modulatory effect on PAG functional connectivity in migraine. Plasma tryptophan concentration showed a diagnosis dependent effect. In healthy control group, there was no correlation between plasma tryptophan concentration and PAG intrinsic connectivity. Meanwhile, in migraine patients increased plasma tryptophan concentration was associated with a reduced functional connectivity between PAG and fusiform gyrus, middle occipital gyrus and cerebellum; and increased connectivity of PAG with superior and superior medial part of frontal gyrus (more precisely with dorsolateral–dlPFC, and dorsomedial prefrontal cortex–dmPFC) in migraine. This PAG functional connectivity pattern that was associated with tryptophan concentration shows opposite direction of connectivity pattern compared to previous studies that investigated PAG connectivity during pain-task^18^, and during resting state fMRI studies in migraineurs with increased anxiety and depressive symptoms^55^ or in a mixed group of migraineurs with and without aura^17^. These studies observed an increased PAG connectivity to nociceptive and somatosensory processing pathways and decreased PAG connectivity to top-down pain and emotion modulating areas that might be counterbalanced by the elevated plasma tryptophan concentration in our study. In agreement with this hypothesis, our episodic migraine subjects without aura and with similar trait-anxiety and depressive symptom scores as controls only showed significantly increased PAG connectivity in the region of the postcentral gyrus corresponding to the head and neck area of sensory homunculus in comparison with healthy controls.

Most of the regions that showed altered PAG-FC among migraineurs after taking into account plasma tryptophan concentration are part of the defence cascade^56^ and play a role in survival salience^12^. One such region is the fusiform gyrus that is functionally connected to PAG and has a main role in evaluating the environment (movements, vocalizations, and faces) to identify potentially dangerous signals^57^. In case of threatening environment PAG activation occurs, while the fusiform gyrus provides top-down inhibitory control on defensive responses to fear when the situation is safe^58^. Previous studies demonstrated both positive and negative connectivity between PAG and fusiform gyrus depending of which PAG subregion was observed^17,59^. In migraine, a recent study reported that during painful stimuli fusiform gyrus showed increased PAG connectivity compared to controls^18^, thus fear cascade may be more active under pain condition in migraineurs. Our results showed that in rest the PAG—fusiform gyrus intrinsic functional connectivity decreases with higher plasma tryptophan concentration. These results might indicate that in absence of any pain there is a decrease in feeling of threat due to less active fear cascade in migraine patients with elevated plasma tryptophan concentration.

In line with this hypothesis, our results showed an increasing dlPFC and dmPFC connectivity with PAG in parallel with higher plasma tryptophan concentration. The dlPFC and dmPFC have integrative roles in selecting and maintaining appropriate emotional behaviour^60^ and also controlling pain perception^61^. For example, contextual fear discrimination, the process which uses previous experiences to evaluate the perceived threats of the current situation, is highly dependent on the connectivity between the dmPFC and PAG^62^. Thus, the strength of this connection is crucial to determine the appropriate defensive or exploratory behaviour. Previous studies separately demonstrated positive PAG connectivity with dmPFC and dlPFC in healthy controls^59^ and migraine patients^17^. But they suggested weaker PAG connectivity with dmPFC and dlPFC in migraine patients compared to controls^17,18,55^ that may contribute to impaired top-down control of pain and fear responses. Based on our results, increasing plasma tryptophan concentration may strengthen PAG connectivity with dmPFC/dlPFC that helps in optimising cortical control on fear cascade.

Another important brain structure in the defence cascade is the cerebellum that has rich anatomical connections to the PAG^63^, serves as an anatomical substrate to the fear-evoked freezing behaviour^56,64^, and has been increasingly implicated in migraine pathophysiology^65,66^. Beside its important role in motor function, cerebellum is involved in encoding of aversive stimuli such as pain and unpleasant images^67^. Previous studies reported a positive functional resting-state correlation between PAG and cerebellum in healthy people^59,68^. Furthermore, migraineurs compared to healthy controls showed decreased functional correlation between PAG and cerebellum during heat pain stimuli^18,67^. Nevertheless in migraineurs, PAG—cerebellum IV-VI connectivity negatively meanwhile PAG—cerebellum crus 1 and IX positively correlated with migraine attack frequency during painful stimulus showing the complexity of the PAG—cerebellum relationship in pain processing^18^. Indeed, based on previous studies, between PAG and cerebellum, direct and indirect connections exist which functions are dependent on the subregions observed^63^. We did not find correlation between migraine indicators and PAG—cerebellum connectivity during resting-state without pain. In our study, increasing plasma tryptophan concentration was associated with decreasing PAG—cerebellum IV–VI intrinsic functional connectivity in migraineurs. That might suggest a decreasing fear cascade activation with increasing plasma tryptophan concentration, but direct comparison with previous findings is difficult because this field is relatively unexplored.

The middle occipital gyrus is part of the multisensory processing brain regions where spatial localisation of visual, tactile and auditory stimuli occurs^69^. It has extensive negative intrinsic functional connectivity with PAG in healthy people based on previous studies^59,68^ and showed increased activity during mind wandering away from pain^70^. However, it also showed greater activity during the activation of the defence cascade by viewing threatening images compared to less arousing stimuli^71^. Solstrand et al.^18^ reported decreased pain-induced PAG-middle occipital gyrus functional connectivity in migraineurs compared to controls and negative correlation between migraine attack frequency and PAG-middle occipital gyrus intrinsic functional connectivity. Contrary to this, we did not find correlation between migraine indicators and PAG-middle occipital gyrus functional connectivity. Thus, although the exact role of the PAG-middle occipital gyrus intrinsic functional connectivity in the defence cascade is not known it can be hypothesised that the decreased intrinsic functional connectivity between these regions, associated with increased plasma tryptophan concentration, may again contribute to decreased pain related fear response.

### Migraine indicators not, but trait-anxiety and depressive symptoms are associated with tryptophan correlated PAG connectivity in migraine

Migraine attack frequency and age at onset although showed positive association with PAG intrinsic functional connectivity in regions previously implicated in migraine (triangular part of inferior frontal gyrus and middle frontal gyrus)^17,18,55^ these connections remained unchanged after correcting for the effect of plasma tryptophan concentration. These results correspond well to previous observations that dietary tryptophan manipulation to increase brain serotonin synthesis was not associated with decreased migraine attack frequency in most of the patients^52,53^.

In contrary to this, the positive correlation between trait-anxiety and PAG connectivity with left dlPFC and dmPFC in migraine patients disappeared after correction for plasma tryptophan (and LNAA) concentration. Impaired defensive behaviour is a main psychopathological root of anxiety disorders and, as we discussed above, PAG–dmPFC connectivity plays a pivotal role in contextual fear discrimination^56,62^. In a recent animal study, Yin et al. demonstrated that the activation of excitatory descending pathway from dmPFC to vlPAG has analgesic and antianxiety effects^72^. Our results might suggest that tryptophan and its metabolites actively modulate the functional coupling of PAG and the dmPFC/dlPFC. Thus, increased strength of PAG–dmPFC/dlPFC connectivity with elevated plasma tryptophan concentration might contribute to analgesic and anxiolytic effect in migraineurs. Accordingly, our migraine patients had episodic migraine with relatively low attack frequency and the mean trait-anxiety symptom scores were in comparable range of controls.

Similarly to trait-anxiety, depressive symptoms showed positive correlation with PAG–dlPFC connectivity in migraine patients that was diminished after correcting for plasma tryptophan concentration. PAG is an active participant in cognitive processes, through its afferent and efferent connections with prefrontal cortex it affects negative emotions and pain related autonomic and behavioural responses^73^. Although PAG–dlPFC connectivity changes has not been investigated in depression but hypoactivity of dlPFC was reported in depressed patients and an increase in its activity after treatment^74^. Our results suggest, that elevated tryptophan concentration by enhancing PAG–dlPFC connectivity may be beneficial in migraineurs by decreasing pain induced negative affect and improving emotion processing^75,76^. Again, we have to emphasize that our migraine subjects had no psychiatric disorders and their depressive symptoms scores were not significantly different from controls. Previous studies demonstrated no mood lowering effect of tryptophan depletion in never-depressed healthy people, but predisposing factors such as high familial risk for depression or remitted depression make people susceptible to depressive symptoms due to tryptophan concentration changes^51^. Our study is in concordance with these observations, in control subjects, tryptophan plasma concentration neither showed significant association with depressive and trait-anxiety symptoms scores, nor influenced PAG connectivity pattern.

In case of depressive symptoms, we also demonstrated negative correlation with PAG connectivity with the right fusiform gyrus and right parahippocampal gyrus that diminished after correction for plasma tryptophan concentration. In a previous study, right fusiform gyrus showed increased intrinsic brain activity in depressed migraine patients compared to non-depressed migraineurs^77^, which is in line with the fear cascade mechanisms we discussed above. Our study suggest that tryptophan might interfere with fear processing through influencing PAG-fusiform gyrus connectivity that can decrease the activity of fear cascade^58^. Regarding parahippocampal gyrus, a positive functional connectivity between PAG-parahippocampal gyrus was found both in migraineurs and in controls^17^, but the exact role of PAG-parahippocampal gyrus connectivity in depressive symptoms is not known yet.

### Limitations

Our study has some important limitations. More women participated in the study than men, but it is in accordance with the disease prevalence sex distribution and all reported results were controlled for age and sex. Plasma tryptophan concentration may have a cyclic variation throughout the menstrual cycle^78^. In our study, there was no significant difference (χ^2^ = 0.071 p = 0.790) between the control (11 women were in the follicular phase, and 8 were in the luteal phase) and migraine group (8 women were in the follicular phase, and 7 were in the luteal phase) regarding the menstruation cycle but more migraine patients used oral contraceptives than controls (2 controls, 6 migraineurs) that could influence the plasma tryptophan level. To ensure that our results are not driven by sex imbalance, we carried out the analysis in sex matched subgroups (27–27 migraineurs vs. controls with 21 female and 6 male participants). However, the extended sex imbalanced sample showed very similar results suggesting that our results are stable, representing differences between migraine patients and controls. Another limitation of the study is the determination of the seed region, that is an important step in every seed-to-voxel connectivity analysis. In the literature, both right and left side coordinates are reported to localise PAG^12^ and in our analysis there is a difference between the connectivity pattern of the two sides, so we decided to separately analyse the right and left PAG seeds. However, results from the analysis with merged PAG seed are reported in the Supplementary information that also supported our major findings and conclusions (Supplementary Tables S8–S10). Furthermore, we measured peripheral potential biomarker, thus we cannot detect the exact biochemical changes in brain areas. However, the repeated measure of plasma tryptophan concentration provided a better estimation of the average tryptophan concentration in the body, and the correction for other LNAA may improve the detection of its effect on brain mechanisms. In addition, combination of resting-state fMRI with biological and psychological data, as in our study, could be a way forward to investigate a complex, multifactorial disorder and identify migraine subgroups with different underlying pathological mechanisms.

## Conclusions

Our study demonstrated a correlation between plasma tryptophan concentration and PAG-FC in migraine for the first time. This correlation was associated with the emotional symptoms of migraine patients but did not interfere with processes that contribute to attack generation or attack frequency. Plasma tryptophan concentration through a more optimal PAG connectivity network might contribute to a better top-down cortical control on contextual fear discrimination and a dampened defence cascade response. These mechanisms might enhance coping strategies in migraine patients. Whether these patients consume tryptophan rich diet as a self-medication or this increased plasma tryptophan concentration is the result of increased stress, as we mentioned above, should be investigated in future studies.

## Methods

### Participants

Eighty-two subjects were included in the study, 34 episodic migraine patients without aura (mean age (*SD*) = 26.53 years (4.70); 28 women) and 48 healthy controls (mean age (*SD*) = 25.69 years (4.05); 29 women). The participants were recruited via university advertisements, newspaper articles and from Headache Clinics. All participants’ mental health was checked using the Mini-International Neuropsychiatric Interview^79^ by senior researchers. Exclusion criteria was having any past or current serious medical, neurologic (except migraine without aura) or psychiatric disorders. Use of any daily medication, except contraceptives was exclusion criteria for both patients and controls. Blood samplings and fMRI scans were carried out if the patients and controls were medication-free for at least 48 h before the examination. In case of migraine patients, blood samplings and fMRI scans were carried out if they were headache-free for at least 48 h before the examination. Only those patients were included into the analyses who were also headache free for at least 24 h after the examination. Both patients and controls refrained from caffeine 4 h before taking blood sample. Participants were all right handed according to the Edinburgh Handedness Inventory^80^. On the fMRI examination day, participants completed the State-Trait Anxiety Inventory (STAI)^81^ and the 20-item Zung Self-Rating Depression Scale^82^ about depressive symptoms.

Episodic migraine without aura was diagnosed by expert neurologists using International Classification of Headache Disorders-III criteria (Headache Classification Committee of the IHS 2013). Patients were asked about the acute migraine medication usually used and clinical indicators of migraine, age of disease onset and frequency of attacks per month (Table 3).

**Table 3:** Clinical data of migraine patients.

Subject	Age of migraine onset (years)	Migraine frequency (per month)	Triptan	Acute migraine medication
1	21	10	Yes	Ibuprofen/paracetamol + caffeine
2	23	5	Yes	Diclofenac, domperidone, ibuprofen
3	17	1.5	No	Metamizole
4	9	1	Yes	Metamizole
5	15	6	Yes	Metamizole, domperidone
6	10	4	No	Diclofenac, domperidone
7	9	6	No	Diclofenac, ibuprofen
8	8	8	No	Ibuprofen, diclofenac, paracetamol + caffeine
9	12	8	Yes	Diclofenac, domperidone
10	16	3	No	Metamizole + caffeine
11	8.5	6	Yes	Domperidone
12	26	2	No	Ibuprofen
13	9	1	No	Ibuprofen, paracetamol + caffeine
14	18	1	No	Metamizole, ibuprofen
15	3	11	Yes	–
16	25	1	No	Ibuprofen
17	14	2	No	Paracetamol
18	15.5	2.5	No	Ketoprofen
19	23	1	No	Metamizole, paracetamol + caffeine
20	14	1.5	No	Ibuprofen
21	25	2	Yes	–
22	15	1.5	No	Ibuprofen
23	14.5	1.5	Yes	Naproxen
24	14.5	4	No	Ibuprofen, diclofenac
25	13.5	1	No	Ibuprofen
26	11	1	No	Diclofenac, metamizole + caffeine
27	6	2	No	Ibuprofen, metamizole, paracetamol + caffeine

Triptan: sumatriptan (50 mg or 100 mg), in one case zolmitriptan.

The study protocol was approved by the Scientific and Research Ethics Committee of the Medical Research Council (Hungary) and conducted in accordance with the Declaration of Helsinki. Each participant gave a written informed consent before entering the study.

### Blood samples

Two separate blood samples were collected at least 28 days apart to evaluate the average tryptophan intake. 74 participants’ (31 migraine patients, 43 controls) first blood samples and 68 participants’ (27 migraine patients, 41 controls) second blood samples were successfully acquired. The blood samples were collected into 3 mL K3EDTA tubes and immediately centrifuged. Plasma samples were frozen and kept at − 80 °C until the assay. LC–MS/MS method already published by Virág et al.^83^ was used for the quantitative determination of total tryptophan and other large amino acids (valine, leucine, isoleucine, phenylalanine, tyrosine) concentration.

### Imaging data acquisition

After the second blood sample collection, participants underwent a 6-min resting-state fMRI session using a 3 T MRI scanner (Achieva 3 T, Philips Medical System). They were instructed to close their eyes, but remain awake. The imaging dataset acquisition parameters of T2*-weighted echo-planar (EPI) pulse-sequence were: repetition time (TR) = 2.500 ms, echo time (TE) = 30 ms, field of view (FOV) = 240 × 240 mm^2^; with 3 × 3 × 3 mm^3^ resolution. High-resolution structural data were acquired before the resting-state acquisition using T1-weighted 3D turbo field echo (TFE) sequence and 1 × 1 × 1 mm^3^ resolution.

### Data analysis

Participants characteristics, self-reported data and amino acid levels were analysed with SPSS (IBM Corp. SPSS Statistics for Windows, Version 25.0). Pearson chi-square test was applied to investigate sex difference between migraine and control groups. Mann–Whitney tests were used to determine any differences between migraine and control groups regarding tryptophan concentration or age. The sum of LNAA affecting tryptophan blood–brain-barrier crossing was calculated (namely tyrosine, phenylalanine, leucine, isoleucine and valine)^27^ and also estimated for any difference between migraineurs and controls. Spearman correlation was used to determine the relationship between age and tryptophan concentration. Wilcoxon signed-rank test was applied to determine any difference between the first and second blood samples. Since, there was no difference in plasma tryptophan concentration between the first and second blood samples, the mean tryptophan concentration was used to characterize the mean tryptophan intake that showed normal distribution. Univariate ANOVA was applied to determine the difference between migraine and control group regarding the mean tryptophan concentration corrected for age, sex and LNAA. Independent t-test was applied to determine whether migraine patients and controls differed in trait-anxiety and depressive symptoms scores. Pearson correlation was calculated to determine the association between tryptophan concentration and anxiety or depressive symptoms. Significance threshold was set at p < 0.05. Two healthy controls did not complete the psychological questionnaires.

### Resting-state fMRI data analysis

State of the art preprocessing steps were carried out on the data according to an optimized pipeline which is shown in detail in Supplementary information.

The final sample for the functional connectivity analysis consisted of 64 subjects (37 controls and 27 migraineurs) with usable biological and resting-state fMRI data. However, to correct for sex imbalance we report the results of a sex-matched case control analysis of 27 migraine and 27 control subjects (21 females and 6 males in each group) in the main manuscript. Results based on the full 64 subject were provided in the Supplementary information.

Seed regions were defined after Mainero et al.^17^ (peak coordinates in MNI space: Left PAG = −2; −28; −6; Right PAG = 4; −28; −6; radius: 3 mm). The fslmaths command of FSL was used to create spherical masks within a 6 mm radius of these two regions for seed selection in a seed-based correlation analysis. For time-series data extraction, followed by voxel-wise connectivity analysis computations NiBabel (v2.3.1)^84^ and NumPy (v1.15.4)^85^ modules of the Python programming language were utilized. The seed-based connectivity map of each subject, based on voxel-wise Pearson correlation with averaged seed region data, was transformed to Z-scores using Fisher transformation.

These Z maps of each individual were used in within- and between-group comparisons in Statistical Parametric Mapping (SPM12) software package (Wellcome Department of Imaging Neuroscience, Institute of Neurology, London, UK; http://www.fil.ion.ucl.ac.uk/spm12/) implemented in Matlab 2016a (Math Works, Natick, MA). First, PAG intrinsic functional connectivity was determined in whole group analysis using one sample t-test. Next, two sample t-test was conducted to compare PAG connectivity between migraine and control groups. Correlation between plasma tryptophan and PAG connectivity in the whole population was determined by including the mean tryptophan concentration as a regressor. Tryptophan correlation with PAG connectivity based on diagnosis was performed by using F-contrast.

To follow up the F-contrast results, the mean plasma tryptophan concentration effect on PAG intrinsic functional connectivity was investigated in the migraine and control group separately using one sample t-tests. In addition, trait-anxiety level (STAI-T) and depressive symptoms (ZUNG) scores and migraine indicators (attack frequency and age of onset) in the migraine group were investigated whether PAG connectivity and these variables show correlation using them as regressors in t-tests to replicate previous findings. Next, tryptophan concentration was used as covariate in order to determine whether it alters the correlation between PAG connectivity and migraine indicators, and trait-anxiety level or depressive symptoms.

All models contained motion correction parameters, age and sex as covariates of no interest. All tryptophan related analyses were corrected for LNAA levels by including plasma LNAA concentration as a regressor of no interest.

An initial threshold of p < 0.001 uncorrected for multiple comparison and at least twenty contiguous voxels^86^ was used in the analyses. All reported results survived family-wise error correction at a cluster-level threshold of p_FWE_ < 0.05.

### Data visualization

For visualization of statistical maps, significant clusters were downloaded from SPM12 program and used as overlay on the MNI 152 template brain in MRIcroGL^87^.

### Ethics statements

The study was approved by the Scientific and Research Ethics Committee of the Medical Research Council (Hungary). Each participant gave a written informed consent before entering the study in accordance with the Declaration of Helsinki.

## Supplementary Information

Supplementary Information.

## Data Availability

The datasets generated during the analysis are available in the Open Science Framework repository (https://osf.io/etj7h/).
