# Supplementary material for: Association of plasma tryptophan concentration with periaqueductal gray matter functional connectivity in migraine patients
Source: Sci Rep. 2022 Jan 14;12:739. doi: 10.1038/s41598-021-04647-0 (PMC8760301; doi:10.1038/s41598-021-04647-0)
Supplement: Supplementary file 1 — Supplementary Information. [file 41598_2021_4647_MOESM1_ESM.pdf]

Supplementary Information

Association of plasma tryptophan concentration with periaqueductal gray matter functional connectivity in migraine patients

Kinga Gecse[1,2], Dóra Dobos[1,2], Csaba Sándor Aranyi[3], Attila Galambos[2,4], Daniel Baksa[1,2], Natália Kocsel[2,4], Edina Szabó[2,4,5], Dorka Papp[2], Dávid Virág[6], Krisztina Ludányi[6], Gyöngyi Kökönyei[1,2,4], Miklós Emri[3], Gyorgy Bagdy[1,7,8], Gabriella Juhasz[1,2]

[1] Department of Pharmacodynamics, Faculty of Pharmacy, Semmelweis University, Budapest, Hungary

[2] SE-NAP2 Genetic Brain Imaging Migraine Research Group, Hungarian Brain Research Program, Semmelweis University, Budapest, Hungary

[3] Division of Nuclear Medicine and Translational Imaging, Department of Medical Imaging, Faculty of Medicine, University of Debrecen, Debrecen, Hungary

[4] Institute of Psychology, ELTE Eötvös Loránd University, Budapest, Hungary

[5] Center for Pain and the Brain (PAIN Research Group), Department of Anesthesiology, Critical Care and Pain Medicine, Boston Children's Hospital, Harvard Medical School, Boston, MA, United States of America

[6] Department of Pharmaceutics, Faculty of Pharmacy, Semmelweis University, Budapest, Hungary

[7] NAP-2-SE New Antidepressant Target Research Group, Hungarian Brain Research Program, Semmelweis University, Budapest, Hungary

[8] MTA-SE Neuropsychopharmacology and Neurochemistry Research Group, Hungarian Academy of Sciences, Semmelweis University, Budapest, Hungary

Corresponding author:

Gabriella Juhasz

Postal address: Department of Pharmacodynamics, Faculty of Pharmacy, Semmelweis University, Budapest, Hungary, 1089 Budapest, Nagyvarad ter 4. Hungary.

Phone: +36-1-4591500/56362

Fax: +36-1-4591494

E-mail: juhasz.gabriella@pharma.semmelweis-univ.hu

Supplementary Table S1. Descriptive characteristics of participants in the sex matched case control design

	Migraine	Control	Test-statistic	p-value
**Sex**	F=21, M=6	F=21, M=6	$\chi^2 = 0$	1
**Age (years)**	25.9 ± 4.6	25.6 ± 4.0	U = 362	p=0.965
**TRP (µg/ml)**				
1.Blood Sample	9.32 ± 2.88	7.12 ± 3.52	U = 136	0.002
2.Blood Sample	9.07 ± 2.66	7.52 ± 2.27	t(2.21)=4.78	0.032
Mean	9.16 ± 2.35	7.33 ± 2.51	F(1,53)=9.467	0.003*
**LNAA (µg/ml)**				
1.Blood Sample	108.79 ± 37.00	116.80 ± 33.62	U=228	0.219
2.Blood Sample	101.67 ± 30.85	115.10 ± 28.80	t(44)=-1.50	0.140
Mean	104.15 ± 28.09	117.45 ± 26.56	F(1,53)=3.886	0.054**
**Zung**	35.27 ± 5.34	33.44 ± 7.05	t(52)=1.065	0.292
**STAI-T**	39.34 ± 8.63	37.63 ± 10.85	t(52)=0.639	0.526
**Age of migraine onset (years)**	14.65 ± 1.2	NA		
**Migraine attack frequency (month)**	3.51 ± 0.57	NA		

Note. Data are expressed as mean value ± SD. The *p* values are based on Pearson chi-square tests for sex, Mann-Whitney U tests for Age, 1. blood sample of TRP and LNAA, and Independent samples t-test for 2. blood sample of TRP and LNAA, STAI-T and Zung. *The difference of mean tryptophan concentration between migraine and control groups was corrected for age, sex and plasma LNAA level using univariate ANOVA.

**The difference of mean LNAA level between migraine and control groups was corrected for age and sex using univariate ANOVA.

LNAA: Large neutral amino-acids, N: Number of participants, F: Female, M: Male, STAI-T: Trait-anxiety level scores, TRP: L-tryptophan plasma concentration, Zung: Depressive symptoms scores, Significance threshold: p<0.05,

Supplementary Table S2. Intrinsic functional connectivity of left and right side of PAG in the sex matched case control design

Seed region	Region	Cluster size (voxel)	Peak T- value	MNI coordinates (x y z)		
Left PAG	R Thalamus	324	4.37	10	-28	-2
	R Precuneus	139	4.85	4	-70	54
Right PAG	R Thalamus	514	19.87	12	-22	10
	L Cerebelum Crus 1	1576	5.42	-40	-52	-34
	L Cerebelum_VI		4.62	-12	-68	-26
	L Cerebelum Crus 2		3.86	-38	-70	-44
	L Vermis VI		3.73	-4	-58	-22
	R Cerebelum VI	428	4.41	26	-62	-30
	R Cerebelum Crus 1		3.60	32	-56	-36
	R Cerebelum VIII		3.49	18	-64	-38

Note. Results are corrected for age and sex. All the listed clusters showed significant positive correlations with PAG, while no negative correlation survived the cluster-level $p_{FWE} < 0.05$ significance threshold.

R: right, L: left, MNI: Montreal Neurological Institute

Supplementary Table S3. Alterations of PAG connectivity in association with migraine frequency, migraine onset, trait-anxiety level and depressive symptoms in migraine patients with and without correction for plasma tryptophan concentration

**Without correction for tryptophan concentration**						**After correction for tryptophan concentration**					
Region	Cluster-size	Peak T-value	MNI-coordinates (x y z)			Region	Cluster-size	Peak T-value	MNI-coordinates (x y z)		
**Attack frequency**						**Attack frequency**					
Left PAG: *Positive correlation*						Left PAG: *Positive correlation*					
R Triangular part of inferior frontal gyrus	181	5.68	50	40	10	R Triangular part of inferior frontal gyrus	232	7.12	50	40	10
R Middle frontal gyrus	141	5.04	24	48	4	R Middle frontal gyrus		3.98	54	34	22
Right PAG: *Positive correlation*						Right PAG: *Positive correlation*					
R Triangular part of inferior frontal gyrus	272	5.4	50	28	26	R Triangular part of inferior frontal gyrus	271	5.47	50	28	26
R Middle frontal gyrus	161	5.21	24	50	4	R Superior frontal gyrus	126	4.07	32	58	14
**Age of onset**						**Age of onset**					
Left PAG: *Positive correlation*						Left PAG: *Positive correlation*					
R Precuneus	197	5.23	14	-44	4	R Lingual gyrus	289	5.45	14	46	4
R Lingual gyrus		4.31	6	-56	4	R Cuneus		4.94	6	66	20
R Cuneus		4.18	6	-64	20						
**Trait-anxiety**						**Trait-anxiety**					
Left PAG: *Positive correlation*						No significant result					
L Middle frontal gyrus	166	5.33	-30	56	28						
Right PAG: *Positive correlation*											
L Middle frontal gyrus	190	5.73	-26	54	30						
L Superior medial frontal gyrus	123	5.09	-4	36	32						
**Depressive symptoms**						**Depressive symptoms**					
Left PAG: *Positive correlation*						Left PAG: *Negative correlation*					
L Middle frontal gyrus	283	6.65	-26	54	28	R Middle cingulate gyrus	206	-4.08	4	-40	42
Left PAG*: Negative correlation*						L Precuneus		-4.44	-10	56	26
R Fusiform gyrus	158	-4.89	26	-40	-18						
R Parahippocampal gyrus		-4.14	30	-30	-16						
Right PAG: *Positive correlation*											
L Middle frontal gyrus	309	6.70	-26	54	30						

*Note.*Significance threshold was cluster-level $p_{FWE}< 0.05$ including at least 20 contiguous voxels. Results are corrected for age, sex and LNAA concentration.
R: right, L: left, MNI: Montreal Neurological Institute

Supplementary Table S4. Association between PAG intrinsic functional resting-state connectivity and plasma tryptophan concentration in migraine patients after correction for trait-anxiety or depressive symptoms.

Region	Cluster-size	Peak T-value	MNI-coordinates (x y z)		
**After correction for trait-anxiety level**					
Left PAG					
Negative correlation					
L Fusiform gyrus	143	- 6.86	-32	-40	-22
Right PAG					
Positive correlation					
L Superior frontal gyrus	155	6.12	20	44	32
L Middle frontal gyrus		4.43	26	38	30
Negative correlation					
L Fusiform gyrus	162	-4.15	-40	-46	-24
**After correction for depressive symptoms**					
Left PAG					
Negative correlation					
L Fusiform gyrus	133	-6.65	-32-	-38	-22
Right PAG					
Positive correlation					
R Superior frontal gyrus	175	6.74	20	44	32
R Superior medial part of Frontal gyrus		4.37	8	56	38
Negative correlation					
L Fusiform gyrus	135	-3.89	-40	-46	-24

Note. Significance threshold was cluster-level $p_{FWE} < 0.05$ including at least 20 contiguous voxels. Results are corrected for age, sex and LNAA concentration. R: right, L: left, MNI: Montreal Neurological Institute

Supplementary Table S5 Descriptive characteristics of participants in the extended unbalanced sample

	1. Blood Sample		Test statistic	p -value
**Total**	74			
**Diagnosis**	Migraine	Control		
**N**	31	43		
**Sex**	F=25, M=6	F=19, M=24	χ^2=4.965	0.026
**Age (years)**	26.71 ± 4.84	25.91 ± 4.06	U=614	0.564
**TRP (µg/ml)**	9.48 ± 2.92	7.85 ± 3.14	U=423	0.008
**LNAA (µg/ml)**	111.49±35.20	116.86±32.40	U=573	0.306
**Age of migraine onset (years)**	14.52 ± 1.14	NA		
**Migraine attack frequency (month)**	3.33 ± 0.50	NA		
	2. Blood Sample			
**Total**	68			
**Diagnosis**	Migraine	Control		
**N**	27	41		
**Sex**	F=21, M=6	F=17, M=24	χ^2 =2.693	0.101
**Age (years)**	26.19 ± 4.73	25.44 ± 3.96	U=515	0.628
**TRP (µg/ml)**	9.07 ± 2.66	7.42 ± 2.1	U=333	0.006
**LNAA (µg/ml)**	101.67±30.85	110.80±27.46	U=410	0.114
**Age of migraine onset (years)**	14.85 ± 1.15	NA		
**Migraine attack frequency (month)**	3.40 ± 0.56	NA		
	Resting-state fMRI			
**Total**	64			
**Diagnosis**	Migraine	Control		
**N**	27	37		
**Sex**	F=21, M=6	F=21, M=16	χ^2=3.058	0.080
**Age (years)**	25.9 ± 4.6	25.7 ± 4.3	U=493	0.929
**TRP (µg/ml)**	9.16 ± 2.35	7.51 ± 2.34	$F(1,63)$=10.005	0.002*
**LNAA (µg/ml)**	103.14±26.69	117.11±23.44	U=360	0.058
**Age of migraine onset (years)**	14.65 ± 1.2	NA		
**Migraine attack frequency (month)**	3.51 ± 0.57	NA		
**STAI-T**	39.34 ±8.63	37.66 ± 10.47 (N = 35)	$t(60)$=0.980	0.503
**ZUNG**	35.27 ± 5.34	33.73 ± 6.72 (N = 35)	$t(60)$=0.674	0.331

Note. Data are expressed as mean value ± SD. The *p* values are based on Pearson chi-square tests for sex, Mann-Whitney U tests for Age, TRP, LNAA, and Independent samples t-test for STAI-T and Zung. *The difference of mean tryptophan concentration between migraine and control groups was corrected for age, sex and plasma LNAA level in the resting state fMRI group using univariate ANOVA. LNAA: Large neutral amino-acids, N: Number of participants, F: Female, M: Male, Significance threshold: p<0.05, STAI-T: Trait-anxiety level scores, TRP: L-tryptophan plasma concentration, Zung: Depressive symptoms scores

Supplementary Table S6. Intrinsic functional connectivity of left and right side of PAG in the extended sample (27 migraineurs and 37 controls)

Seed region	Region	Cluster size (voxel)	Peak T- value	MNI coordinates (x y z)		
Left PAG	L Thalamus	1435	4.44	-12	-26	-8
	R Thalamus		4.35	12	-18	12
	L Caudate		4.15	-10	4	12
	L Superior frontal gyrus		4.90	-26	62	10
	L Middle frontal gyrus		3.68	-24	60	16
	L Superior frontal gyrus, medial part		3.64	-12	66	10
	L Cerebelum Crus2	426	4.54	-40	-68	-42
	L Cerebelum_VI		4.24	-28	-58	-36
	L Cerebelum Crus1		4.16	-42	68	-32
	L Cuneus	316	4.17	-2	-68	24
	L Precuneus		3.21	-12	-66	38
	R Middle frontal gyrus	277	4.10	42	6	56
			3.88	36	12	46
	L Vermis_VI	268	4.22	-2	-58	-24
	L Cerebelum_VI		4.15	-8	-66	-22
	R Vermis_VII		3.74	4	-74	-24
Right PAG	R Thalamus	1567	4.78	12	-18	12
	L Thalamus		3.73	-14	-26	8
	L Cerebelum Crus2	2654	4.99	-36	-68	-40
	L Cerebelum_VI		4.68	-28	-60	-34
	L Cerebelum_IV_V		4.25	-6	-60	-22
	L Superior frontal gyrus	167	4.42	-26	62	10
	L Middle frontal gyrus		3.47	-26	48	16
	R Caudate	215	4.09	10	6	6
	R Pallidum		4.02	22	0	6
	L Precuneus	323	4.21	-4	-70	30
	L Cuneus		4.12	-2	-68	22
	R Middle frontal gyrus	237	4.18	44	26	32

Note. Results are corrected for age and sex. All the listed clusters showed significant positive correlations with PAG, while no negative correlation survived the cluster-level $p_{FWE} < 0.05$ significance threshold.

R: right, L: left, MNI: Montreal Neurological Institute

Supplementary Table S7 Significantly different association between PAG intrinsic functional resting-state connectivity and plasma tryptophan concentration in the extended unbalanced sample (27 migraine patients compared to 37 controls)

Region	Cluster size (voxel)	Peak F-value	MNI coordinates (x y z)		
**Left PAG**					
R Middle Occipital gyrus	1020	21.64	28	-92	20
L Middle Occipital gyrus	944	19.07	-20	-88	20
L Superior Occipital gyrus		17.39	-16	-86	30
L Fusiform gyrus	916	20.98	-36	-52	-18
L Cerebellum IV-V		20.83	-6	-64	-6
R Fusiform gyrus	334	14.67	34	-38	-24
**Right PAG**					
L Fusiform gyrus	146	25.94	-34	-66	-12
L Cerebellum VI		14.5	-22	-62	-14
L Middle Occipital gyrus	123	20.61	-36	-76	8

Note. Significance threshold was cluster-level $p_{FWE}< 0.05$ including at least 20 contiguous voxels. Results are corrected for age, sex and LNAA concentration. R: right, L: left, MNI: Montreal Neurological Institute

Supplementary Table S8 Significantly different association between PAG intrinsic functional resting-state connectivity and plasma tryptophan concentration in migraine patients compared to controls using PAG as a single seed

Region	Cluster size (voxel)	Peak F-value	MNI coordinates (x y z)		
L Fusiform gyrus	1060	31.18	-44	-70	-18
L Cerebellum IV-V		24.06	-6	-64	-6
R Fusiform gyrus	1176	23.14	30	-46	-16
R Middle Occipital gyrus		23.79	28	92	20
L Middle Occipital gyrus	696	20.54	-28	-84	15
L Superior Occipital gyrus		17.44	-14	-96	24

Note. Significance threshold was cluster-level pFWE< 0.05 including at least 20 contiguous voxels. Results are corrected for age, sex and LNAA concentration. MNI: Montreal Neurological Institute

Supplementary Table S9 Significant association between PAG intrinsic functional resting-state connectivity and plasma tryptophan concentration in migraine patients using a single seed for PAG

Region	Cluster size (voxel)	Peak T-value	MNI coordinates (x y z)		
Negative correlation					
L Fusiform gyrus	718	-6.76	-32	-48	-18
R Fusiform gyrus	191	-6.10	28	-44	-18
R Cerebellum VI		-4.65	34	-44	-26
L Middle Occipital gyrus	210	-5.38	-24	-84	14
Positive correlation					
R Superior medial part of Frontal gyrus	224	5.05	10	54	36
L Superior medial part of Frontal gyrus	199	4.93	-2	34	34
L Superior Frontal gyrus	183	6.11	-18	48	36

Note. Significance threshold was cluster-level pFWE< 0.05 including at least 20 contiguous voxels. Results are corrected for age, sex and LNAA concentration. MNI: Montreal Neurological Institute

Supplementary Table S10 Alterations of PAG connectivity in association with migraine frequency, migraine onset, trait-anxiety level and depressive symptoms in migraine patients with and without correction for plasma tryptophan concentration, using PAG as a single seed

**Without correction for tryptophan concentration**						**After correction for tryptophan concentration**					
Region	Cluster-size	Peak T-value	MNI-coordinates (x y z)			Region	Cluster-size	Peak T-value	MNI-coordinates (x y z)		
**Attack frequency**						**Attack frequency**					
Positive correlation						Left PAG: *Positive correlation*					
R Middle frontal gyrus	162	5.03	24	50	4	R Triangular part of inferior frontal gyrus	286	6.29	50	40	10
R Triangular part of inferior frontal gyrus	273	5.17	50	40	10	R Middle frontal gyrus		4.41	54	34	22
**Age of onset**						**Age of onset**					
Positive correlation						Left PAG: *Positive correlation*					
R Lingual gyrus R	179	4.93	14	-46	4	R Lingual gyrus	225	5.04	14	-46	4
R Precuneus		4.14	12	-64	24						
**Trait-anxiety**						**Trait-anxiety**					
Positive correlation											
L Middle frontal gyrus	213	5.58	-26	54	30	*No significant results*					
L Superior medial frontal gyrus	129	5.13	-4	36	32						
**Depressive symptoms**						**Depressive symptoms**					
Positive correlation											
L Middle frontal gyrus	314	6.73	-26	54	28						
Negative correlation						*No significant results*					
R Fusiform gyrus	146	-5.49	26	-40	-18						
R Hippocampus		-4.28	34	-26	-10						

Note. Significance threshold was cluster-level $p_{FWE} < 0.05$ including at least 20 contiguous voxels. Results are corrected for age, sex and LNAA concentration.
MNI: Montreal Neurological Institute

Preprocessing pipeline

Freesurfer (version 5.3) (http://surfer.nmr.mgh.harvard.edu/) was used to perform intensity non-uniformity correction and intensity normalization on the T1-weighted images. The Freesurfer workflow also segmented the structural images to extract the brain volume and to separate white matter and gray matter tissues from cerebrospinal fluid (CSF). Spatial normalization was performed with the ANTs registration tool [1], which transformed the extracted brain volumes to match the 2 mm isovoxel brain template in MNI152 space provided by the Montreal Neurological Institute (MNI). The first four frames of the fMRI time-series were deleted from further processing to remove variance introduced while magnetization stabilized. Primary motion correction was performed using the MCFLIRT [2] utility of the FMRIB's Software Library (FSL version 6.0), by moving every image with rigid-body transformation to the first volume of the time-series. The six movement parameters were completed with the movement at the previous time point and with the squared value of each obtained parameter [3]. These 24 time-invariant variables were used at later steps to reduce motion artefacts in the data. For spatial standardization the functional images were coregistered with the corresponding subject’s structural scan with the FSL linear registration tool, then transformed them into MNI152 space using the same transformation we normalized the T1 images with. CompCor technique [4] was investigated to compute the first five principal components of fMRI time-series within the white matter and CSF, which are considered to describe uninteresting variance regarding cortical activation. Then spatial filtering was applied with the FSL application SUSAN [5], using a Gaussian-kernel of 6 mm full-width at half maximum. Further signal correction was performed with the independent component analysis based ICA-AROMA [6], and we linearly regressed the 24 parameters derived from primary motion correction and the 5 CompCor components from the data. Four subjects were excluded due to excessive movements defined as frame-wise relative RMS displacement > 0.25 mm in more than 20 volumes [7]. Finally, temporal band-pass filtering was applied between 0.009 Hz and 0.08 Hz to remove noise and eliminate signal drifting effects.

References

1 Avants, B. B., Epstein, C. L., Grossman, M. & Gee, J. C. Symmetric diffeomorphic image registration with cross-correlation: evaluating automated labeling of elderly and neurodegenerative brain. *Medical image analysis* **12**, 26-41, doi:10.1016/j.media.2007.06.004 (2008).

2 Jenkinson, M., Bannister, P., Brady, M. & Smith, S. Improved optimization for the robust and accurate linear registration and motion correction of brain images. *Neuroimage* **17**, 825-841, doi:10.1016/s1053-8119(02)91132-8 (2002).

3 Friston, K. J., Williams, S., Howard, R., Frackowiak, R. S. & Turner, R. Movement-related effects in fMRI time-series. *Magnetic resonance in medicine* **35**, 346-355, doi:10.1002/mrm.1910350312 (1996).

4 Behzadi, Y., Restom, K., Liau, J. & Liu, T. T. A component based noise correction method (CompCor) for BOLD and perfusion based fMRI. *Neuroimage* **37**, 90-101, doi:10.1016/j.neuroimage.2007.04.042 (2007).

5 Smith, S. M. & Brady, J. M. SUSAN—A New Approach to Low Level Image Processing. *International Journal of Computer Vision* **23**, 45-78, doi:10.1023/A:1007963824710 (1997).

6 Pruim, R. H. R. *et al.* ICA-AROMA: A robust ICA-based strategy for removing motion artifacts from fMRI data. *Neuroimage* **112**, 267-277, doi:10.1016/j.neuroimage.2015.02.064 (2015).

7 Ciric, R. *et al.* Benchmarking of participant-level confound regression strategies for the control of motion artifact in studies of functional connectivity. *NeuroImage* **154**, 174-187, doi:https://doi.org/10.1016/j.neuroimage.2017.03.020 (2017).
